# Design of scalable metalens array for optical addressing

**DOI:** 10.1007/s12200-022-00035-2

**Published:** 2022-08-04

**Authors:** Tie Hu, Xing Feng, Zhenyu Yang, Ming Zhao

**Affiliations:** School of Optical and Electronic Information, Huazhong University of Science and Technology, Wuhan, 430074 China

**Keywords:** Metalens array, Optical addressing, Scalability

## Abstract

Large-scale trapped-ion quantum computers hold great promise to outperform classical computers and are crucially desirable for finance, pharmaceutical industry, fundamental chemistry and other fields. Currently, a big challenge for trapped-ion quantum computers is the poor scalability mainly brought by the optical elements that are used for optical addressing. Metasurfaces provide a promising solution due to their excellent flexibility and integration ability. Here, we propose and numerically demonstrate a scalable off-axis metalens array for optical addressing working at the wavelength of 350 nm. Metalens arrays designed for *x* linearly polarized and left circularly polarized light respectively can focus the collimated addressing beam array into a compact focused spot array with spot spacing of 5 μm, featuring crosstalk below 0.82%.

**Graphical Abstract:**

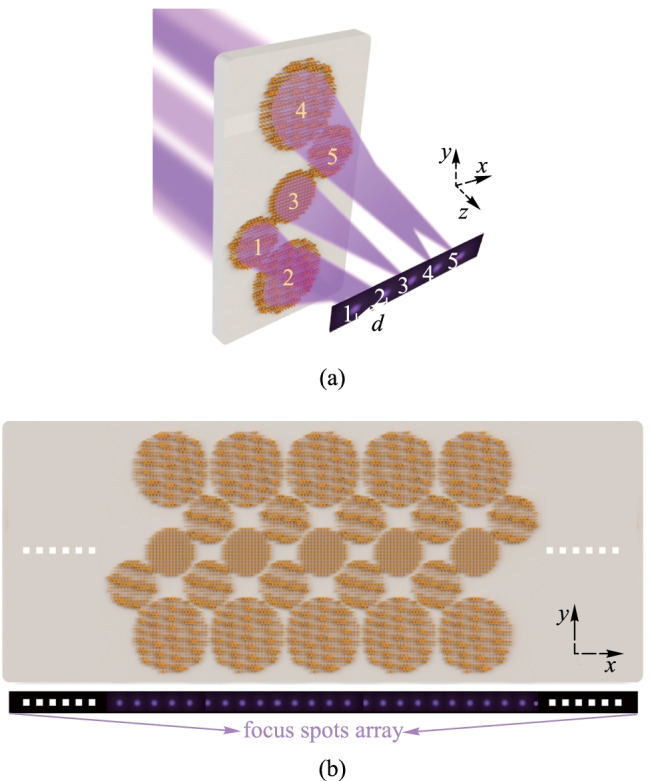

## Introduction

Quantum computers employ qubits that are the quantum superposition of traditional bits 0 and 1, and are expected to outperform classical computers. Trapped-ion quantum computers arouse much research interest due to their advantages over other types of practical quantum computers. However, it is challenging to scale up the number of the trapped-ion qubits while maintaining the ability to control them individually with high operation fidelities [1]. The primary restrictions of current trapped-ion quantum computers result from the free-space optical elements used in the optical addressing system [2]. Optical addressing, a technology to focus and align individual addressing beams onto quantum particles, needs integrated, miniatured, and flexible focusing optical elements to realize precise manipulation over quantum states of individual trapped ion and neutral atom [3]. Previously, many related pioneering works have been reported, such as refractive lens group [4, 5], micromirrors based on micro-electromechanical systems (MEMS) [6, 7], microfabricated Fresnel lens arrays [8] and diffractive mirrors interfaced with reconfigurable planar waveguide circuits [9, 10], and focusing grating couplers [2, 11–13]. Existing technologies are limited by the lack of scalability and face challenges in obtaining good focusing properties, such as diffraction-limited focusing spot size, small focusing spot spacing, low crosstalk, and high efficiency.

Generally speaking, smaller focused spots are more advantageous for suppressing crosstalk between neighboring trapped ions, thus leading to better operation fidelities. According to Abbe’s diffraction limit theory [14], the shorter the working wavelength of the optical focusing element, the smaller the spot radius of the focused spot. Additionally, focused spots with smaller spot radiuses allow quantum logic gates of a given interaction time to use lasers with a lower magnitude of power [11]. Therefore, compact focusing optical elements working in the ultraviolet (UV) have more advantages when used for optical addressing. In trapped-ion quantum computers, ions are typically confined about 30–100 μm above the surface electrode in a vacuum by Coulomb forces [15]. In this article, we aim to design a compact scalable optical focusing element used for optical addressing of linear trapped ions chain, with spot spacing of about 5 μm, working distance of about 30 μm, and spot radius within 0.75 μm.

Metasurfaces, composed of planar subwavelength-scale meta-antenna arrays, have shown versatile capabilities in manipulating amplitude, phase, polarization, and frequency of light at the subwavelength resolution [16]. Also, metasurfaces can be integrated with various functional materials, e.g., dyes, nonlinear materials, liquid crystals, etc., to realize dynamic manipulation [17–19]. Recently, metasurfaces have become an unprecedented platform for realizing numerous compact devices [20–25], such as polarization converters, meta-holograms, and metalenses. Especially, metalenses have boosted a wide range of applications including optical trapping, full-color achromatic imaging, polarization imaging, and microscopic imaging due to their compact size, planar structure, and compatibility with CMOS processing [26–30].

Previously, intensive studies focused on on-axis metalens working in the visible and the near-infrared band. Yet few works studied the ultraviolet UV off-axis metalenses and melalens array. Compact metalenses working at the UV band are crucially important for lithography, imaging, spectroscopy, and quantum computing [31]. Metalens array can generate focused spot array with a compact configuration and has found applications in a wide range of fields from full-color light field imaging [32], optical multiparameter detection [33–35], to the generation of a multiphoton quantum source [36]. On the other hand, the off-axis metalens can modify the position of the focus, thus adding a design freedom compared to the on-axis metalens. Notably, existing designs of metalens array can only generate a spot array with spacing equal to the distance of two neighboring metalenses, while metalens array composed of both on-axis metalenses and off-axis metalenses can achieve an arbitrarily arranged spot array.

In this article, we propose and numerically demonstrate a niobium pentoxide (${\text{Nb}}_{{2}} {\text{O}}_{{5}}$) scalable metalens array (SMA) for optical addressing at the wavelength of 350 nm. The SMA can focus collimated addressing beam array into a chain-arranged focused spot array, with spot spacing of 5 μm, crosstalk below 0.82%, and working distance of about 30 μm. SMAs for *x* linearly polarized (*x*-LP) and left circularly polarized (LCP) light are designed to realize addressing and coherent manipulation of different types of trapped ions. The design might be helpful to promote the development of integrated trapped-ion quantum computers and increase the number of trapped-ion qubits.

## Design of scalable metalens array (SMA)

### Structure of SMA

**Fig. 1:**
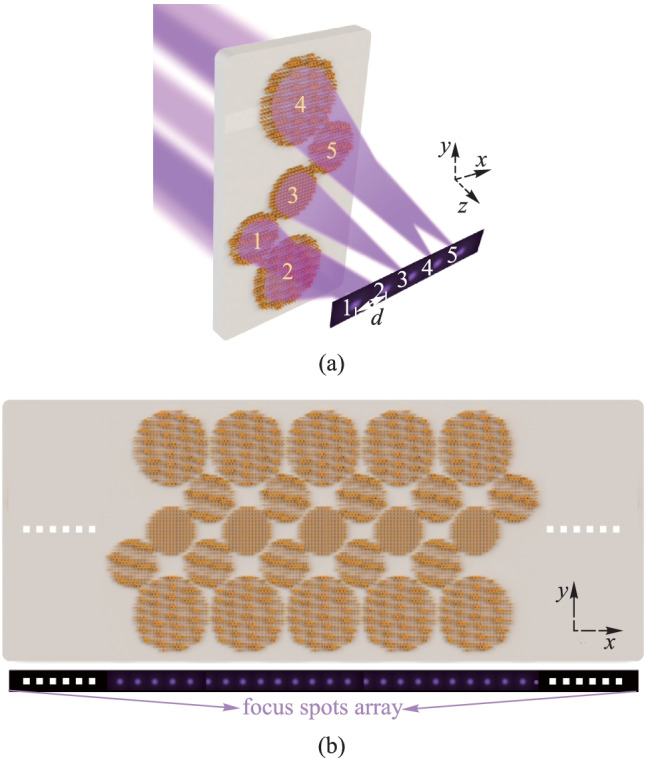
Schematic of the device structure. **a** Diagram of a metalens molecule. **b** Diagram of SMA. The bottom figure shows the focused spot array

The principle of the proposed SMA is illustrated in Fig. 1. The SMA can produce a chain-arranged focused spot array with a uniform spot spacing when the incoming addressing beam array is normally incident from the fused silica substrate. Here, to match the requirements of the designed SMA, the incoming addressing beam array should be arranged in a “Z” shape. For optical addressing, each focused spot should be accurately aligned to the exact trapped ion. And the spot spacing between the center of the focused spot is chosen to match the equilibrium positions of the ion array. Figure 1b shows that the SMA consists of periodical metalens molecules with a period of *d* × *n*, where *d* is the spot spacing and *n* is the number of metalenses in one molecule. Here we take the center of metalens 3 as the coordinate origin, the arrangement direction of the focused spot array as the *x*-axis, and the direction of light propagation as the *z*-axis As depicted in Fig. 1a, a metalens molecule is composed of five metalenses spatially arranged in a “Z” shape, and each metalens corresponding to an exact focused spot is composed of nanopillars on the fused silica substrate.

Detailly, Fig. 1a shows that metalenses 1, 2, 4, and 5 should adopt the off-axis design, while metalens 3 is an on-axis metalens. The combination of both off-axis metalenses and on-axis metalens is used for two reasons: Firstly, the combination of off-axis metalens and on-axis metalens enables the generation of a compact focused spot array in an arbitrarily arranged shape, which means that the arrangement of the metalens array can be different from the arrangement of its focused spot array. e.g., for a metalens array arranged in a “Z” shape, the arrangement of the focused spot array can be a chained shape. Secondly, according to Abbe’s diffraction limit theory, the ideal diffraction limit of a metalens is $\lambda /(2\mathrm{NA})$, where $\lambda$ is the working wavelength, NA is the numerical aperture of a metalens. Considering that we aim to design a metalens array working at 350 nm that can generate a chain-arranged focused spot array with working distance of about 30 μm, spot radius within 0.75 μm, the minimum radius of on-axis metalens is 7.2 μm. If an on-axis metalens array is adopted, the minimum spot spacing is then 14.4 μm, which is much larger than the requirement of 5 μm.

### Nanopillar design

**Fig. 2:**
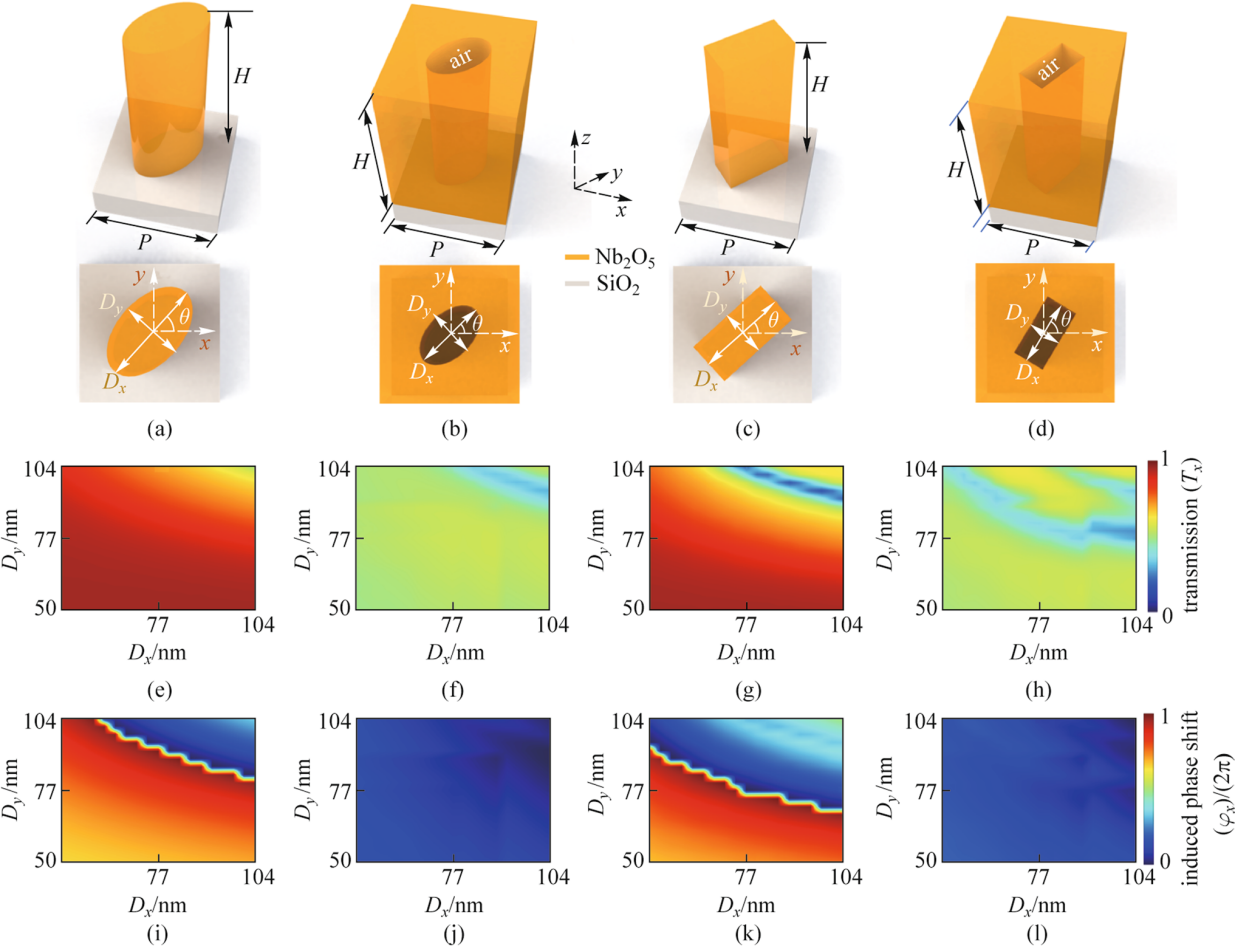
Schematics of the dielectric or air-hole nanopillars and the corresponding transmittances and induced phase-shift under the normal incidence of the *x*-LP plane wave at the wavelength of 350 nm. **a**–**d** are the 3D structure and top-view of four types of nanopillars. **e**–**h** are the transmittances $T_{x}$ and **i**–**l** are the phase-shift ${\boldsymbol{\varphi }}_{x}$ of the nanopillars with different values of *D*_*x*_ and *D*_*y*_. The dielectric or air-hole nanopillars have an elliptical/rectangular cross-section with a long axis and short axis of $D_{x}$ and $D_{y}$; $\theta$ is the rotation angle of the long axis relative to the *x-*axis

Figures 2a–d show the design of elliptical and rectangular ${\text{Nb}}_{{2}} {\text{O}}_{{5}}$ or air-hole nanopillars. The refractive index of ${\text{Nb}}_{{2}} {\text{O}}_{{5}}$ is 2.76 + 0.02i at the wavelength of 350 nm [37]. In general, the electromagnetic response of an anisotropic nanopillar can be described by a Jones matrix [38]:

1 $$M = R( - \theta )\left[ {\begin{array}{*{20}c} {{\text{e}}^{{{\text{i}}\varphi_{x} }} } & 0 \\ 0 & {{\text{e}}^{{{\text{i}}\varphi_{y} }} } \\ \end{array} } \right]R(\theta ),$$

2 $$R(\theta ) = \left[ {\begin{array}{*{20}c} {\cos \theta } & {\sin \theta } \\ { - \sin \theta } & {\cos \theta } \\ \end{array} } \right],$$

where $\varphi_{x}$, $\varphi_{y}$ denote the phase of output light wave under the normal incidence of *x*-LP and *y* linearly polarized (*y*-LP), respectively; $\theta$ is the azimuth angle of nanopillars relative to the *x*-axis; $R(\theta )$ is the rotation matrix.

If the nanopillar is rotated with an angle of $0^{ \circ }$ and normally illuminated by the *x*-LP light which can be described by a Jones vector $\left[ {1 \,\space 0} \right]^{\text T}$, the Jones vector of output light can be written as

3 $$E_{{{\text{out}}}} = M\left[ {\begin{array}{*{20}c} 1 \\ 0 \\ \end{array} } \right] = \left[ {\begin{array}{*{20}c} 1 & 0 \\ 0 & 1 \\ \end{array} } \right]\left[ {\begin{array}{*{20}c} {{\text{e}}^{{{\text{i}}\varphi_{x} }} } & 0 \\ 0 & {{\text{e}}^{{{\text{i}}\varphi_{y} }} } \\ \end{array} } \right]\left[ {\begin{array}{*{20}c} 1 & 0 \\ 0 & 1 \\ \end{array} } \right]\left[ {\begin{array}{*{20}c} 1 \\ 0 \\ \end{array} } \right] = \left[ {\begin{array}{*{20}c} {{\text{e}}^{{{\text{i}}\varphi_{x} }} } \\ 0 \\ \end{array} } \right],$$

4 $$\varphi_{x} = \frac{{2{\uppi }}}{\lambda }n_{{{\text{eff}}}} H,$$

where $\lambda$ is the working wavelength; *H* is the height of the nanopillars. Each nanopillar operates as an optical waveguide, where its effective refractive index ${n}_{{\rm{eff}}}$ can be modified by adjusting the long axis $D_{x}$ and short axis $D_{y}$ of nanopillars. Hence, according to Eq. (4), the phase ${\varphi }_{x}$ of the transmitted field can be changed by modifying the ${n}_{{\rm {eff}}}$ of the nanopillars [20].

The four types of nanopillars (namely the elliptical, rectangular, dielectric or air-hole nanopillars), all with period of *P* = 180 nm and height of *H* = 350 nm, are simulated using the finite-difference time-domain (FDTD) method. Periodic boundary conditions are applied in both *x* and *y* directions, while perfectly matched layers boundary conditions are used in the *z* direction. Figures 2e–h and i–l show the transmittance and induced phase-shift of the nanopillars with different values of *D*_*x*_ and *D*_*y*_ when these nanopillars are under the normal incidence of the *x*-LP plane wave. The transmittance and induced phase-shift can be represented by matrices of $T_{x}$ and ${{\varphi }}_{x}$. It is clear that the combination of the four types of nanopillars can cover the whole phase-shift range of 0 − 2π, which is unfulfillable if using only one type. For simplicity, the transmittance $T_{y}$ and induced phase-shift ${{\varphi }}_{y}$ for the incident *y*-LP plane wave can be obtained by transposing the transmittance $T_{x}$ and induced phase-shift ${{\varphi }}_{x}$.

If the nanopillar is illuminated by circularly polarization (CP) light under normal incidence, as the Jones vector of CP light is $\left[ {1 \space \pm{\text{i}}} \right]^{{\text{T}}}$, the Jones vector of output light can be written as

5 $$E_{{{\text{out}}}} = T\left[ {\begin{array}{*{20}c} 1 \\ { \pm {\text{i}}} \\ \end{array} } \right] = \frac{{{\text{e}}^{{{\text{i}}\varphi_{x} }} + {\text{e}}^{{{\text{i}}\varphi_{y} }} }}{2}\left[ {\begin{array}{*{20}c} 1 \\ { \pm {\text{i}}} \\ \end{array} } \right] + \frac{{{\text{e}}^{{{\text{i}}\varphi_{x} }} - {\text{e}}^{{{\text{i}}\varphi_{y} }} }}{2}\exp ( \pm {\text{i}}2\theta )\left[ {\begin{array}{*{20}c} 1 \\ { \mp {\text{i}}} \\ \end{array} } \right],$$

where + and – denote right circularly polarized (RCP) and LCP incident light, respectively. The transmitted field comprises two orthogonal CP components. The first term corresponds to the component that has the same polarization as the incident light (co-polarization term), and the second one is a cross-polarization term with an additional geometric phase of ± 2$\theta$. Therefore, the full phase coverage of 0 − 2π can be obtained if the nanopillars are rotated from $0^{ \circ }$ to $180^{ \circ }$. Here $\left| {{{({\text{e}}^{{{\text{i}}\varphi_{x} }} - {\text{e}}^{{{\text{i}}\varphi_{y} }} )} \mathord{\left/ {\vphantom {{({\text{e}}^{{{\text{i}}\varphi_{x} }} - {\text{e}}^{{{\text{i}}\varphi_{y} }} )} 2}} \right. \kern-\nulldelimiterspace} 2}} \right|^{2}$ is defined as the polarization conversion efficiency of the nanopillars.

### Off-axis metalens design

The phase profile $\varphi^{(i)}$ of the *i*th metalens following the hyperbolic off-axis phase distribution is given by

6 $$\varphi^{(i)} (x,y) = {\frac {2\uppi }{\lambda}}\left[ f^{(i)} - \sqrt {(x - d_{x}^{(i)} )^{2} + (y - d_{y}^{(i)})^{2} + (d_{z}^{(i)})^{2}} + \varphi_{\rm {c}} \right],$$

where

7 $$f^{(i)} = \sqrt {(d_{x}^{(i)} )^{2} + (d_{y}^{(i)} )^{2} + (d_{z}^{(i)} )^{2} } .$$

$f^{(i)}$ and $d_{z}^{(i)}$ are the focal length and working distance of ***i***th metalens; $(d_{x}^{(i)} ,d_{y}^{(i)} ,d_{z}^{(i)} )$ is the location of the *i*th focused spot relative to the center of the *i*th metalens; *λ* is the working wavelength, and $\varphi_{{\text{c}}}$ is the reference phase of the center of the *i*th metalens. The optical performance of the focused spot can be flexibly manipulated by optimizing $(d_{x}^{(i)} ,d_{y}^{(i)} ,d_{z}^{(i)} )$.

**Fig. 3:**
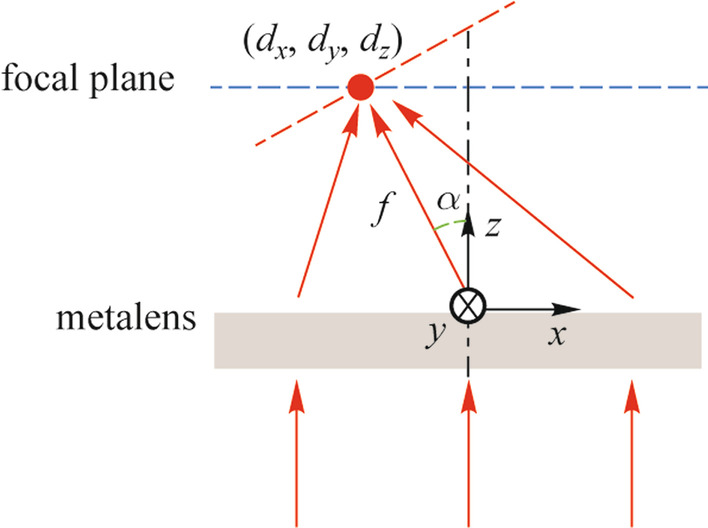
Schematic illustration of light focusing by the off-axis metalens. The black dashed line denotes the focal plane, which is perpendicular to the *z*-axis

Figure 3 shows the schematic illustration of the light focusing by the off-axis metalens, which focuses the normally incident plane wave on a spot whose position is given by $(d_{x} ,d_{y} ,d_{z} )$. The numerical aperture (NA) of an off-axis metalens can be expressed by [39]

8 $${\text{NA}} = \sin \left( {\frac{\beta }{2}} \right),$$

9 $$\beta = \left\{ {\arctan (\tan \alpha + {R \mathord{\left/ {\vphantom {R {d_{z} }}} \right. \kern-\nulldelimiterspace} {d_{z} }}) - \arctan (\tan \alpha - {R \mathord{\left/ {\vphantom {R {d_{z} }}} \right. \kern-\nulldelimiterspace} {d_{z} }})} \right\},$$

where $\alpha$ is the oblique focusing angle; *R* is the radius of the metalens.

For optical addressing used in trapped-ion quantum computers, fully polarized beams are generally required to control the state of the trapped ions. For instance, linearly polarized or elliptically polarized individual addressing beam arrays are needed to control the trapped ${}^{43}\text {Ca}^{ + }$ ion qubits, while for ${}^{171}\text {Yb}^{ + }$ ion qubits, circularly polarized addressing beams are used to realize specific gate operations. Therefore, both SMAs are designed for the *x*-LP and LCP light respectively. Briefly, the SMA is designed for the *x*-LP incidence following the propagation phase principle, while the SMA designed for the LCP incidence is based on the geometric phase [33]. For metalenses working under the normally incident *x*-LP light wave, the nanopillars are designed to realize the smallest average phase error of the transmitted near field with Eq. (6). However, for metalenses working under the normally incident LCP light wave, the rectangular dielectric nanopillar ($D_{x}$ = 104 nm, $D_{y}$ = 59 nm) with the polarization conversion efficiency (62.8%) is selected as the basic unit. Here, the imperfect conversion efficiency is due to the small optical loss of the ${\text{Nb}}_{{2}} {\text{O}}_{{5}}$ nanopillar.

In a metalens molecule, the design of metalenses 1 and 4 follow those of metalenses 5 and 2, respectively because of the geometric symmetry. Thus, only metalenses 2, 3, and 5 need to be designed. The radiuses of metalenses 2, 3, and 5 are 12.5, 8, and 8 μm, respectively. And the working distances of metalenses 2, 3, and 5 are 30.9, 32.1, and 31.7 μm, respectively.

In the simulation, the near-field distribution was obtained by the FDTD method. While the far-field calculation was performed using plane wave expansion and chirped Z-transform to reduce the simulation time [40]. Table 1 summarizes some metrics used in this paper.

**Table 1:** Definitions of metalens’s metrics

Crosstalk	Polarization extinction ratio	Encircled power
${{P_{\rm {N}} } \mathord{\left/ {\vphantom {{P_{\rm {N}} } {P_{{\rm {foc}}} }}} \right. \kern-\nulldelimiterspace} {P_{{\rm {foc}}} }} \times 100\%$	${{P_{{\rm {cross}}} } \mathord{\left/ {\vphantom {{P_{{\rm {cross}}} } {P_{{\rm {co}}} }}} \right. \kern-\nulldelimiterspace} {P_{{\rm {co}}} }} \times 100\%$	${{P_{{\rm {foc}}} } \mathord{\left/ {\vphantom {{P_{{\rm {foc}}} } {P_{{\rm {in}}} }}} \right. \kern-\nulldelimiterspace} {P_{{\rm {in}}} }} \times 100\%$

$P_{\rm {N}}$ is the noise power of the target metalens that results from scatterings of other metalenses in one specific metalens molecule; $P_{\text {foc}}$ is the power $P_{{\rm {3FWHM}}}$ of the target metalens in one specific metalens molecule; $P_{{\rm {cross}}}$ and $P_{{\rm {co}}}$ are respectively the power $P_{{\rm {3FWHM}}}$ of the target metalens under the cross-polarization and co-polarization incidence; $P_{{\rm {in}}}$ is the power incident onto the target metalens [41]; $P_{{\rm {3FWHM}}}$ is the power within a circle whose diameter equals three times full-width at half maximum (FWHM) of the intensity distribution at the focal plane

## Results and discussion

### Results of SMA for the *x*-LP incidence

**Fig. 4:**
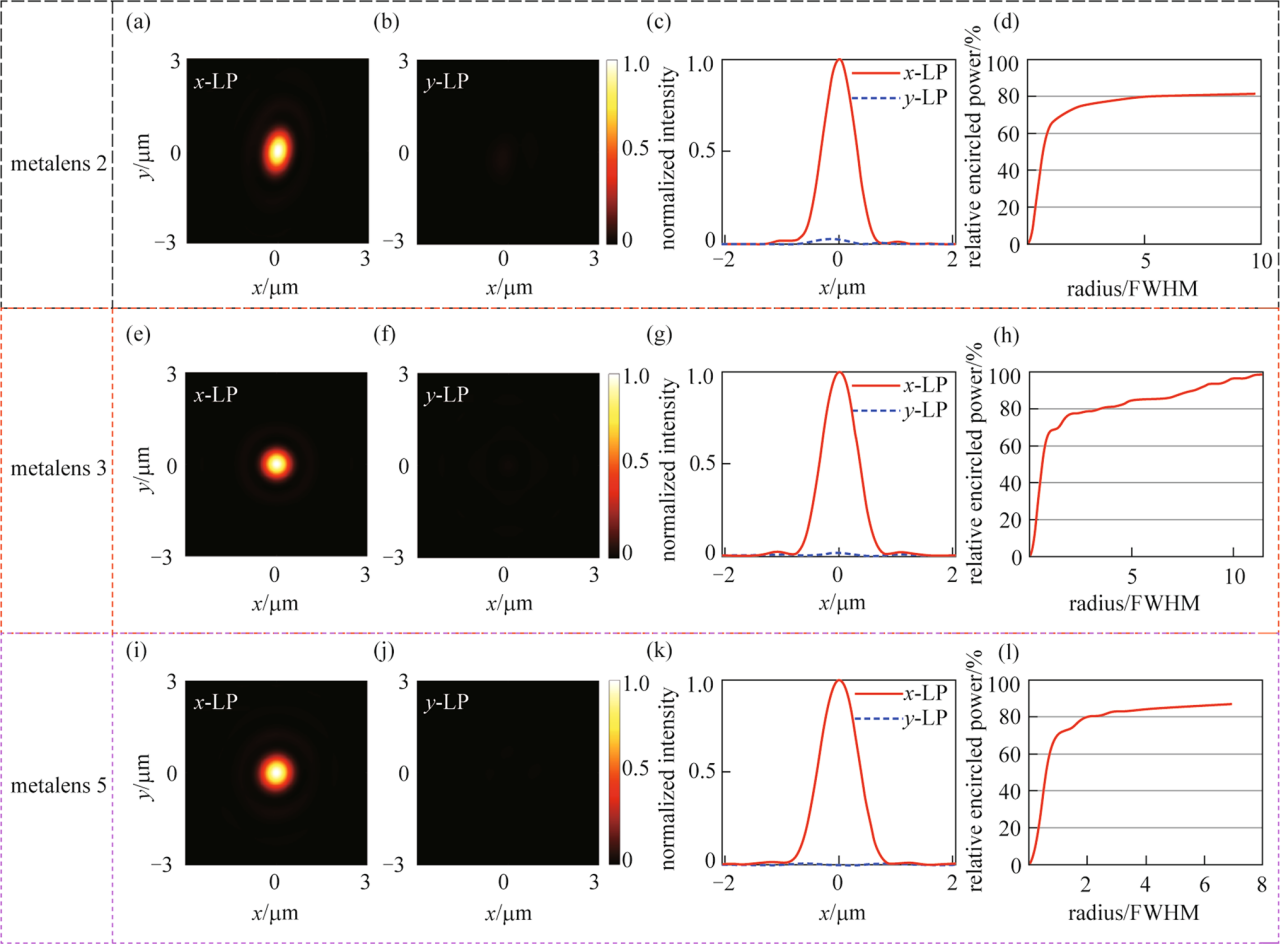
Characterizations of the individual *x*-LP metalenses 2, 3, and 5. **a**, **e**, and **i** Normalized intensity distributions of the focal plane under the *x*-LP incidence. **b**, **f**, and **j** Normalized intensity distributions of the focal plane under the *y*-LP incidence. **c**, **g**, and **k** Normalized intensity distributions along the *x*-axis. **d**, **h**, and **l** Relative encircled power versus the relative radius of the focal plane

Metalenses 2, 3, and 5 in one metalens molecule designed for the *x*-LP light are characterized in Figs. 4. The normalized intensity distributions at the focal plane of metalenses 2, 3, and 5 (illuminated by the *x*-LP light) are respectively shown in Figs. 4a, e, and i. These results verify that the metalenses can focus the collimated *x*-LP light into tightly optical spots. Under the *y*-LP incidence, the normalized intensity distributions at the focal plane of metalenses 2, 3, and 5 are respectively shown in Figs. 4b, f, and j. And these results demonstrate that metalenses 2, 3, and 5 can well scatter the incoming *y*-LP light. For visualization, Figs. 4c, g, and k, respectively show the intensity distributions along the *x*-axis at the focal plane. The relative encircled power of metalenses 2, 3, and 5 are shown respectively in Figs. 4d, h, and l. These results demonstrate that the designed metalenses for *x*-LP incident light have functionalities of a polarizer and a focusing lens at the same time, which is preferred in controlling the trapped ${}^{43}\text {Ca}^{ + }$ ion qubits for optical addressing applications. Taking metalens 2 as an example, the polarization extinction ratio is 13.97 dB calculated from the results shown in Figs. 4a and b. The spot radius is 0.64 μm calculated from the results of Fig. 4c, which is smaller than the reported results of other optical focusing elements for optical addressing applications, to the best of our knowledge. Since there are discrepancies in the utilization of focusing efficiency to characterize a metalens, we would rather use the relative encircled power instead [41]. Figure 4d indicates that more than 80% of the power is confined within a circle with a radius of seven times FWHM of intensity distribution at the focal plane. Here we note that the encircled power can be improved by using materials (${\text{Si}}_{{3}}\text {N}_{4}$, ${\text{HfO}}_{{2}}$, etc.) of less optical loss and optimizing the nanopillars for higher transmittance. Results for metalenses 3 and 5 are similar.

**Fig. 5:**
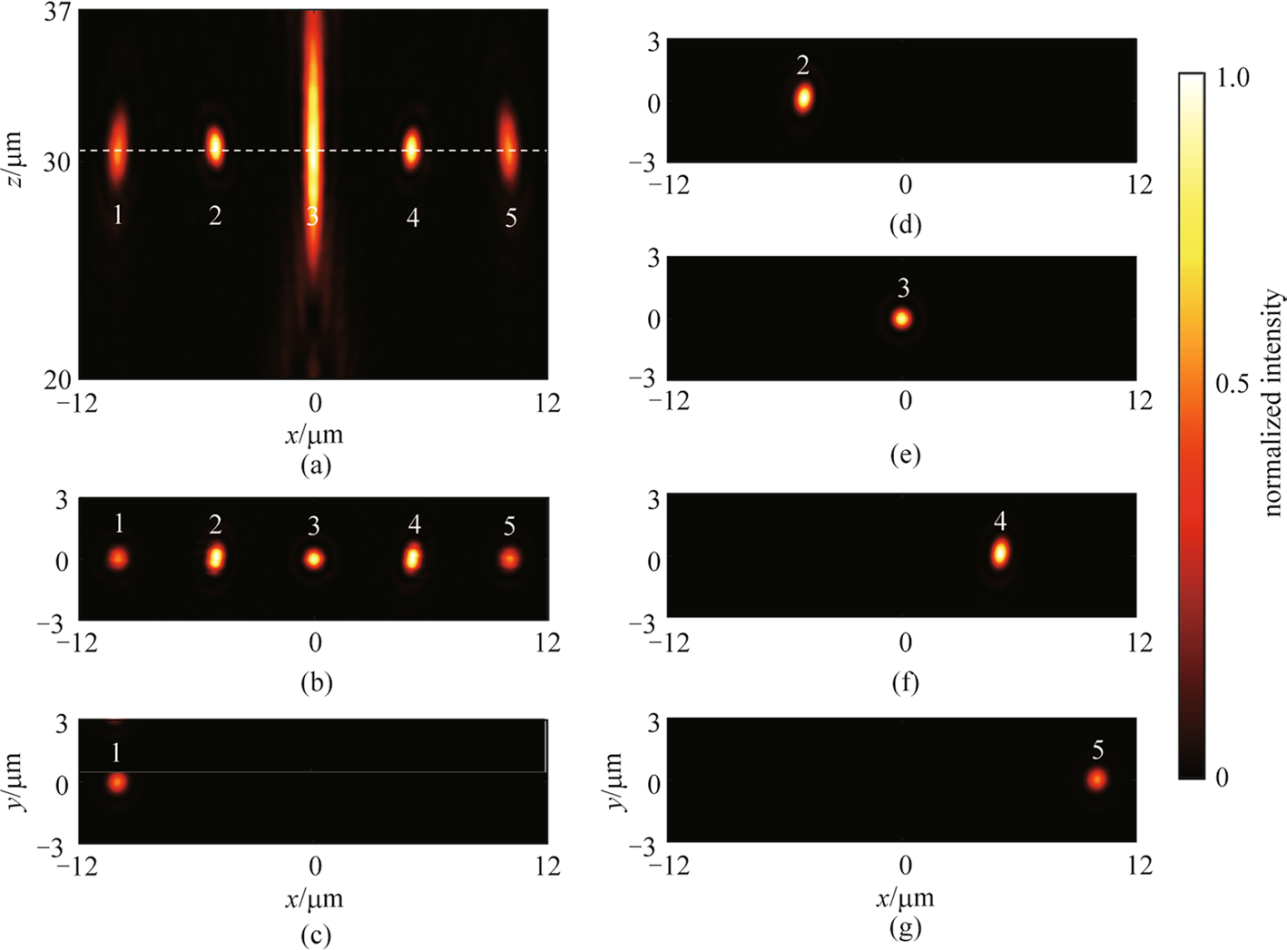
Characterization of the metalens molecule for the *x*-LP light wave. **a** Normalized intensity distribution at the *xz* section. **b** Normalized intensity distribution at the focal plane when all the metalenses are illuminated. **c**–**g** Normalized intensity distributions at the focal plane when each target metalens is respectively illuminated. All the intensity distributions are normalized by the maximum intensity of the metalens molecule designed for the *x*-LP light

Furthermore, the performance of the *x*-LP metalens molecules is illustrated in Fig. 5. The normalized intensity distribution at the *xz* plane is shown in Fig. 5a, which indicates that the five metalenses have the same working distance of 30.00 μm. Figures 5b is the normalized intensity profile at the focal plane when all five metalenses are illuminated. It is observed that the five focused spots are arranged in a chain with uniform spot spacing. Notably, the elliptical focused spots 1, 2, 4, and 5 are due to asymmetric phase distributions of the corresponding metalenses. Figures 5c–g depict the normalized intensity profiles at the focal plane when each target metalens is illuminated, respectively. It is found that the crosstalk of metalens 2 resulting from the scattering of other metalenses in one metalens molecule is 0.25%, whereas they are 0.15%, and 0.19% for metalenses 3 and 5, respectively. Low crosstalk might enhance the operation fidelity of quantum computers.

### Results of SMA designed for the LCP light

**Fig. 6:**
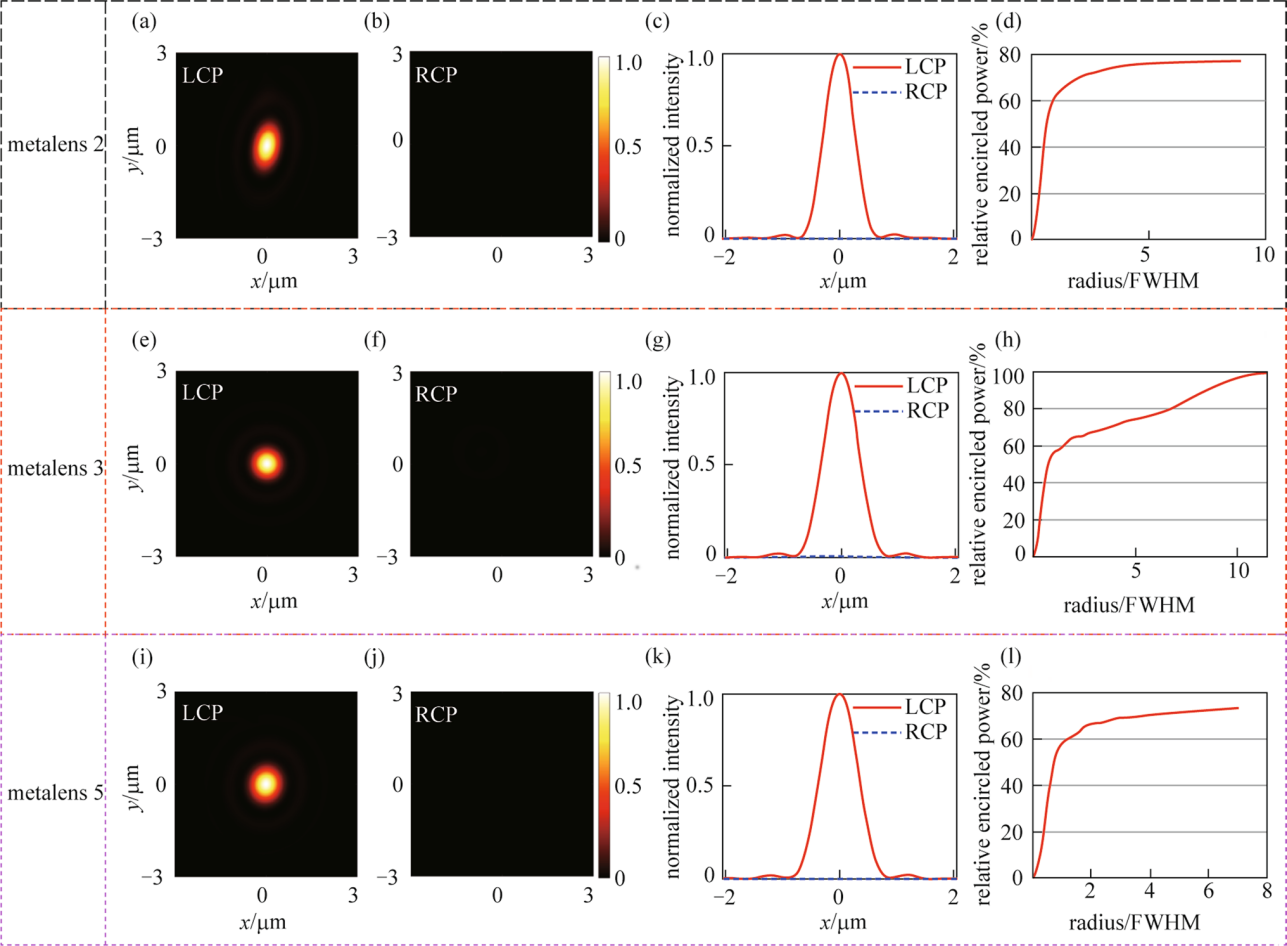
Characterizations of the individual metalens 2, 3, and 5 for the LCP plane wave. **a**, **e**, and **i** Normalized intensity distributions at the focal plane under the LCP incidence. **b**, **f**, and **j** Normalized intensity distributions at the focal plane under the RCP incidence. **c**, **g**, and **k** Normalized intensity distributions along the *x*-axis at the focal plane. **d**, **h**, and **l** Relative encircled efficiency vs. relative radius of the focused spot

Figure 6 shows simulated results of metalenses 2, 3, and 5 designed for the LCP plane wave. The normalized intensity distributions at the focal plane of metalenses 2, 3, and 5 are respectively shown in Figs. 6a, e, and i, when these metalenses are illuminated by LCP incidence. While Figs. 6b, f, and j, respectively show the normalized intensity distributions at the focal plane of metalenses 2, 3, and 5, which are illuminated by the RCP light. Then the polarization extinction ratios of the metalenses 2, 3, and 5 are 27.42 (calculated from the results of Figs. 6a and b), 15.86 (calculated from the results of Figs. 6e and f), and 25.63 dB (calculated from the results of Figs. 6i and j), respectively. These results demonstrate that the metalenses designed for the LCP incident light have good polarization sensitivity, which is preferred in controlling the trapped ${}^{171}\text {Yb}^{ + }$ ion qubits for optical addressing applications. That means the designed metalenses can generate tightly focused spots when the collimated LCP plane wave illuminates the metalenses, while the incident RCP light will be diverged by the metalenses. Figures 6c, g, and k, respectively show the normalized intensity distributions along the *x*-axis at the focal plane of metalenses 2, 3, and 5 under LCP and RCP incidence, where the spot radiuses are calculated to be 0.61, 0.71, and 0.75 µm, respectively. Figures 6d, h, and l indicate that nearly 70% of the powers are confined within a circle with radiuses of seven times FWHM of intensity distribution at the focal plane. These results demonstrate that the metalenses designed for LCP light have good focusing performance.

**Fig. 7:**
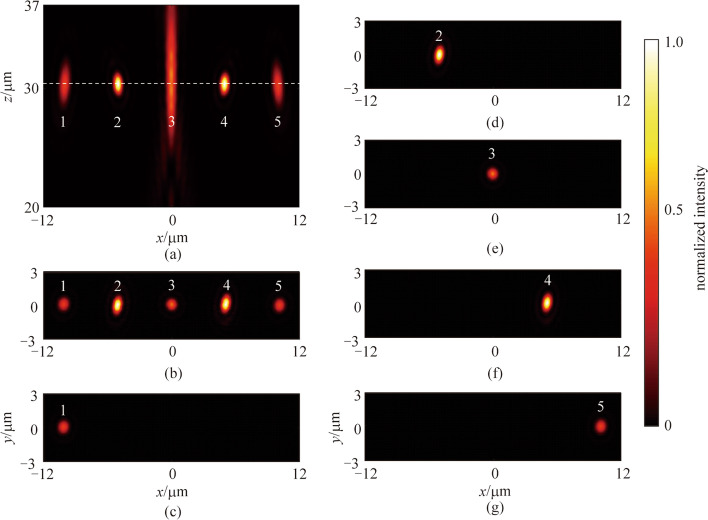
Characterization of the metalens molecule designed for the LCP light. **a** Normalized intensity distribution at the *xz* section. **b** Normalized intensity distribution at the focal plane when all the metalenses are illuminated. **c**–**g** Normalized intensity distributions of the focal plane when each target metalens is illuminated, respectively. All the intensity distributions are normalized by the maximum intensity of the metalens molecule designed for the LCP light

To characterize the overall performance of the metalens molecule designed for LCP incident light, the normalized intensity distributions at the *xz* plane and the focal plane are shown in Figs. 7a and b–g, respectively. It can be observed that a focused spot array is produced which consists of five chain-arranged focused spots, with the same working distance of 30.00 μm and uniform spot spacing. In one metalens molecule designed for LCP incident light, the crosstalks of metalenses 2 from the scattering of other metalenses is 0.82%, while the crosstalks of metalenses 3 and metalenses 5 are respectively 0.07% and 0.06%.

## Conclusions

In conclusion, the *x*-LP and LCP SMAs are designed for optical addressing at the UV band. These SMAs can focus the two-dimensional addressing beam array into chain-arranged focused spot arrays, each with spot spacing of 5 μm, and working distance of about 30 μm, featuring crosstalk below 0.82%. In the practical applications of optical addressing, the designed SMA can generate an arbitrary number of focused spots by choosing the suitable number of the metalens molecules, thus metalens array with good scalability can be obtained. The relative encircled power of the metalens arrays can be improved by using materials (${\text{Si}}_{{3}}\text {N}_{4}$, ${\text{HfO}}_{{2}}$, etc.) with less optical loss and optimized by selecting nanopillars with higher transmittance. Considering the versatile dispersion manipulation ability of metasurfaces, a multiwavelength or achromatic SMA can be realized, which might find an application in the optical addressing of hybrid trapped-ion quantum computers.
